# Transgenic Centipedegrass (*Eremochloa ophiuroides* [Munro] Hack.) Overexpressing *S*-Adenosylmethionine Decarboxylase (SAMDC) Gene for Improved Cold Tolerance Through Involvement of H_2_O_2_ and NO Signaling

**DOI:** 10.3389/fpls.2017.01655

**Published:** 2017-09-22

**Authors:** Jianhao Luo, Mingxi Liu, Chendong Zhang, Peipei Zhang, Jingjing Chen, Zhenfei Guo, Shaoyun Lu

**Affiliations:** ^1^State Key Laboratory for Conservation and Utilization of Subtropical Agro-bioresources, Guangdong Engineering Research Center for Grassland Science, College of Life Sciences, South China Agricultural University Guangzhou, China; ^2^Department of Grassland Science, College of Agronomy, Hunan Agricultural University Changsha, China; ^3^College of Grassland Science, Nanjing Agricultural University Nanjing, China

**Keywords:** antioxidants, centipedegrass, cold tolerance, hydrogen peroxide, nitrate reductase, nitric oxide, polyamines, *S*-adenosylmethionine decarboxylase (SAMDC)

## Abstract

Centipedegrass (*Eremochloa ophiuroides* [Munro] Hack.) is an important warm-season turfgrass species. Transgenic centipedgrass plants overexpressing *S-adenosylmethionine decarboxylase* from bermudagrass (*CdSAMDC1*) that was induced in response to cold were generated in this study. Higher levels of *CdSAMDC1* transcript and sperimidine (Spd) and spermin (Spm) concentrations and enhanced freezing and chilling tolerance were observed in transgenic plants as compared with the wild type (WT). Transgenic plants had higher levels of polyamine oxidase (PAO) activity and H_2_O_2_ than WT, which were blocked by pretreatment with methylglyoxal bis (guanylhydrazone) or MGBG, inhibitor of SAMDC, indicating that the increased PAO and H_2_O_2_ were a result of expression of *CdSAMDC1*. In addition, transgenic plants had higher levels of nitrate reductase (NR) activity and nitric oxide (NO) concentration. The increased NR activity were blocked by pretreatment with MGBG and ascorbic acid (AsA), scavenger of H_2_O_2_, while the increased NO level was blocked by MGBG, AsA, and inhibitors of NR, indicating that the enhanced NR-derived NO was dependent upon H_2_O_2_, as a result of expression *CdSAMDC1*. Elevated superoxide dismutase (SOD) and catalase (CAT) activities were observed in transgenic plants than in WT, which were blocked by pretreatment with MGBG, AsA, inhibitors of NR and scavenger of NO, indicating that the increased activities of SOD and CAT depends on expression of *CdSAMDC1*, H_2_O_2_, and NR-derived NO. Our results suggest that the elevated cold tolerance was associated with PAO catalyzed production of H_2_O_2_, which in turn led to NR-derived NO production and induced antioxidant enzyme activities in transgenic plants.

## Introduction

Polyamines are important plant regulators involving in plant growth, development and adaptation to environmental stresses (Minocha et al., 2014; Shi and Chan, 2014; Liu et al., 2015). Putrescine (Put), spermidine (Spd), and spermine (Spm) are three major plant polyamines. Put is synthesized from arginine, catalyzed by arginine decarboxylase, *N*-carbamoylputrescine amidohydrolase, and agmatine iminohydrolase sequentially, or from ornithine, catalyzed by ornithine decarboxylase. Spd is synthesized from decarboxylated *S*-adenosylmethionine (dcSAM) and Put, catalyzed by Spd synthase, while dcSAM is formed from *S*-adenosylmethionine (SAM), catalyzed by SAM decarboxylase (SAMDC). Spm is synthesized from Spd and Put, catalyzed by Spm synthase (SPMS, **Supplementary Figure S1**; Liu et al., 2015). Polyamines are oxidized to produce H_2_O_2_ catalyzed by polyamine oxidase (Liu et al., 2015). H_2_O_2_ is demonstrated to induce *NITRATE REDUCTASE1* (*NIA1*) expression which is responsible for nitric oxide (NO) production (Rockel et al., 2002; Bright et al., 2006; Lu et al., 2014). H_2_O_2_ and NO are signaling in multiple physiological processes including adaptation to environmental stresses (Desikan et al., 2004; Miller et al., 2008; Zhao et al., 2009; Farnese et al., 2016; Niu and Liao, 2016; Sewelam et al., 2016; Singh et al., 2016).

Polyamines accumulate in plants in response to drought (Li et al., 2015), salinity (Tassoni et al., 2008), and cold stress (Kovács et al., 2010; Chen et al., 2013), while plant tolerance to drought and cold is increased by exogenous application of polyamines (Shi et al., 2013; Peng et al., 2016). Plant tolerance to abiotic stress may be modified by regulation of polyamine synthesis For example, overexpression of *ADC*, *ODC*, and *SPDS* (Roy and Wu, 2001; Kumria and Rajam, 2002; Kasukabe et al., 2004; He et al., 2008) result in enhanced abiotic stress tolerance in transgenic plants, while knock-out or down-regulation of *ADC*, *SPDS*, and *SPMS* genes decreases tolerance (Kasinathan and Wingler, 2004; Yamaguchi et al., 2007; Cuevas et al., 2008). SAMDC is a key enzyme for Spd and Spm formation. Transgenic rice and tobacco plants down-regulating *SAMDC* expression have decreased Spd and Spm levels along with reduced tolerance to drought, salinity, and cold (Moschou et al., 2008; Chen et al., 2014), while transgenic plants overexpressing *SAMDC* had enhanced Spd and Spm levels along with elevated tolerance to drought (Waie and Rajam, 2003), salinity (Waie and Rajam, 2003; Hao et al., 2005; Wi et al., 2006), cold (Hao et al., 2005; Wi et al., 2006), and heat (Cheng et al., 2009). However, there is no report to modulate abiotic stress tolerance in turfgrass by overexpressing *SAMDC* gene.

Centipedegrass is a warm-season turfgrass species with excellent adaptation to low pH and poor soil, thick sod formation, and uniform and aggressive growth. It is a low maintenance grass and requires infrequent mowing due to its slow-growing habit. Thus it is commonly used in soil conservation, residential lawns, and recreational turf in tropical and subtropical regions and a grazing-purpose grass for low-input grassland systems in Japan (Hanna and Liu, 2003; Hirata et al., 2016). Centipedegrass can be potentially used for phytoremediation due to its capacity to transport heavy metals from roots to shoots and leaves (Li et al., 2016). Low temperature is a major environmental factor limiting the plantation of centipedegrass. Reactive oxygen species (ROS) is accumulated in plants under low temperature conditions when the absorbed light energy cannot used by CO_2_ assimilation as a result of inhibition of Calvin–Benson cycle enzymes. These accumulated ROS may result in oxidative damages of photosynthetic apparatus if it could not be scavenged effectively. Antioxidant defense system protects plant against the oxidative damages by scavenging ROS for maintenance of ROS homeostasis in plant cells under stress conditions (Miller et al., 2008). By using gamma-ray radiation, a chilling-tolerant mutant was selected in our laboratory. The mutant maintained higher levels of antioxidants and polyamines during chilling stress compared with the WT, suggested that polyamines and antioxidants are associated with chilling tolerance in centipedegrass (Chen et al., 2013). However, centipedegrass has low genetic diversity (Hanna and Liu, 2003; Harris-Shultz et al., 2012), which limits improvement of centipedegrass. Plantlet generation and *Agrobacterium*-mediated transformation in centipedegrass have been established in our laboratory (Liu et al., 2008, 2012). The objectives of this study were to increase cold tolerance in centipedegrasss by modulating polyamine synthesis through overexpressing a *SAMDC* gene from bermudagrass and investigate whether H_2_O_2_ and NO were involved in the improved cold tolerance in transgenic plants.

## Materials and Methods

### Plant Growth Conditions and Treatments

Centipedegrass plants and a common bermudagrass (*Cynodon dactylon*) that was used in our previous study (Lu et al., 2008) were planted in 15-cm diameter plastic pots containing a mixture of peat and perlite (3:1, v/v) in a greenhouse for 2 months, with temperature ranging from 25 to 30°C, irrigating daily and fertilizing once a week with 15N–6.6P–12.5K fertilizer. Bermudagrass plants were placed in a growth chamber with a 12-h photoperiod under light of 200 μmol m^-2^ s^-1^ at 6°C for 4 days for cold treatment for analysis of *CdSAMDC1* expression, while centipedegrass plants were used for physiological and molecular measurements. For treatment with chemicals, leaf fragments of transgenic plants and WT were placed in deionized water for 1 h to eliminate the potential wound stress, and then placed in beakers containing 1 mM methylglyoxal *bis* (guanylhydrazone) or MGBG, 1 mM ascorbic acid (AsA), 1 mM NaN_2_, 100 mM tungstate, or 200 mM 2-phenyl-4,4,5,5-tetramethylimidazoline-1-oxyl3-oxide (PTIO) under light of 80 mmol photons m^-2^ s^-1^ for 12 h, while those treated with deionized water for 12 h under the same condition served as a control. After treatments the leaf fragments were sampled and immediately frozen under liquid N_2_ for further analysis.

### Cloning of *CdSAMDC1*

Total RNA was isolated from bermudagrass leaves by using TRIzol reagent (Life Technologies, Carlsbad, CA, United States) according to the manufacturer’s protocol. First-strand cDNA was synthesized from 1 μg of total RNA in a 20-μl reaction mixture, using M-MLV reverse transcriptase and Oligo (dT)_18_ primer. For amplification of *CdSAMDC1*, polymerase chain reaction (PCR) was conducted in a reaction mixture containing the first-strand cDNA as the template, primers SAMDC_F (CCTGCTCCAATGGCTGTTCT) and SAMDC_R (CCCGTCTTACTCATCAAGCACTC), and *KOD*-*Plus* DNA polymerase (TOYOBO, Osaka, Japan).

### Generation of Transgenic Centipedegrass Plants

Embryogenic calli were induced from sterilized mature seeds of centipedegrass and cultivated as previously described (Liu et al., 2008). The embryogenic callus was transformed using *Agrobacterium tumefaciens* strain EHA105 harboring pCAMBIA-35S-*CdSAMDC1* construct as described previously (Liu et al., 2012). the calli were placed on callus induction medium without selection pressure for 1-week after co-cultured for 3 days, followed by placing on selection medium containing hygromycin B (50 mg l^-1^) for 8-week. The hygromycin-resistant calli were subjected for regeneration on regeneration medium containing hygromycin (50 mg l^-1^), illuminated with a 16 h photoperiod (80 μmol m^-2^ s^-1^). The regenerated shoots were transferred to half strength of MS medium containing sucrose for rooting. The plantlets were transferred to soil in 15-cm plastic pots growing in a greenhouse at temperatures of 30/25°C (day/night) under natural light.

### DNA Blot Hybridization

One gram of leaves was used for extract genomic DNA using the hexadecyltrimethylammonium bromide (CTAB) method. DNA samples (20 μg) were digested overnight with *Hind*III, separated by electrophoresis on 0.8% agarose gel, and transferred to Hybond XL nylon membrane (Amersham, GE Healthcare Limited, Buckinghamshire, United Kingdom), sequentially. The coding sequence of *hpt* was labeled as DNA probes for hybridization using a PCR digoxigenin (DIG) probe synthesis kit (Roche Diagnostics, Basel, Switzerland). The DNA filter was washed sequentially with 2× SSC, 0.1% SDS; 1× SSC, 0.1% SDS for 10 min at room temperature; and 0.5× SSC, 0.1% SDS for 15 min at 65°C after prehybridization and hybridization. Hybridization signals were detected using a Lumivision PRO (TAITEC, Saitama, Japan).

### Real-time Quantitative RT-PCR

Total RNA was extracted as described above. One μg of total RNA was used for synthesis of first-strand cDNA, using the PrimeScript RT reagent Kit With gDNA Eraser (Takara Bio, Inc., Otsu, Shiga, Japan). After diluted for 50-fold, the cDNA was used as template for real-time quantitative RT-PCR (qRT-PCR) analysis in a total of 10 μl PCR reaction, containing 15 ng of cDNA, 200 nM each of forward and reverse primers, and 5 μl SYBR Premix *Ex Taq* (Takara Bio, Inc., Otsu, Shiga, Japan), with three technical replicates and two biological replicates. A parallel reaction to amplify *actin1* was used to normalize the amount of template. The PCR primers include: *CdSAMDC1* forward primer ZG5547 (5′-CGCCATCGAAGCAATAGAAAAC-3′) and reverse primer ZG5548 (5′-CCGCGGCCAAGCAGGAA-3′);*actin1* forward primer ZG1551 (5′-TCTTGCTGGTCGTGACTTGACGG-3′) and reverse primer ZG1552 (5′-ACCTGGCCATCAGGCAGCTCAT-3′). The primers were designed using the software tool Beacon Designer (Premier Biosoft International, Palo Alto, CA, United States), and the primer specificity was validated by melting profiles and showed a single product specific melting temperature. All PCR efficiency was above 95%.

### Determination of Cold Tolerance

The temperature that resulted in 50% lethal (LT_50_) was calculated using a fitted model plot for evaluation of freezing tolerance (Pennycooke et al., 2008; Guo et al., 2014). Freezing tolerance was also evaluated by survival rate. Stolons were cut into segments with one node, and placed in a beaker incubating in a Programmable Freezer (model: Polystat cc1 & k6, Huber Unit, Offenburg, Germany), following freezing treatment by decreasing temperature from 25 to 0°C linearly within 6 h, and maintained at 0, -2, and -3°C for 1 h respectively. After thawing overnight at 0°C, the stolons were planted in soil for vegetative propagation until new shoots were regenerated. The regenerated plants were counted and survival rate were calculated. The experiments were performed three times using 40 stolons segments each line per replicate. For assessment of chilling tolerance, centipedegrass plants were moved into a growth chamber at 6°C with a 12-h photoperiod under light of 200 μmol m^-2^ s^-1^ for 30 days as described previously (Chen et al., 2013). Net photosynthetic rate (*A*) was measured by using a LI-6400P Portable Photosynthesis System (LI-COR, Inc., Lincoln, NE, United States) (Chen et al., 2013).

### Determination of H_2_O_2_

Leaves were stained in 1 mg ml^-1^ of 3,3-diaminobenzidine (DAB) solution for 1 h, followed by decoloring in boiling ethanol (95%) for 20 min before photography (Orozco-Cárdenas and Ryan, 1999). In another case, H_2_O_2_ was determined spectrophotometrically as previously described (Zhou et al., 2006).

### Determination of Polyamines

Free polyamines were extracted and measured as described previously (Chen et al., 2013). Leaves (0.5 g) were extracted in 5 ml of 5% (v/v) cold perchloric acid (PCA) and incubated on ice for 1 h. The homogenate was centrifuged at 20,000 ×*g* for 30 min. Aliquots (0.5 ml) of supernatant were mixed with 1 ml of 2 M NaOH and 7 μl of benzoyl chloride and incubated at 37°C for 20 min in dark for benzoylation. The benzoylated polyamines were extracted to diethyl ether, resuspended in 1 ml of mobile phase solution (64% methanol in an isocratic elution), and filtered (4.5 μm filter) before HPLC analysis. Twenty μl of sample was injected into a Waters chromatographic system (Waters, Mildford, MA, United States), supplied with a C18 column (4.6 mm × 250 mm), and detected at 254 nm using a 2487 dual UV detector (Waters, Milford, MA, United States). Polyamine levels were calculated based on standard curves of commercial standards in combination with a recovery of the extraction procedure.

### Determinations of Polyamine Oxidase (PAO) Activity

Polyamine oxidase was extracted in 0.1 M phosphate buffer (pH 7.0), and the activity was measured as described previously (Chen et al., 2014). The reaction was initiated and incubated at 30°C for 30 min after addition of 20 μl of 20 mM Spd or Spd into reaction mixture (3 ml) that was consisted of 2.50 ml of 0.1 M phosphate buffer (pH 7.0), 0.1 ml of horseradish peroxidase (250 units), 0.2 ml of coloring solution (25 μl *N, N*-dimethylaniline and 10 mg 4-aminoantipyrine were dissolved in 100 ml of 0.1 M phosphate buffer, pH 7.0) and 0.2 ml enzyme extract or inactivated enzyme (by heating the enzyme for 20 min in a boiling water bath) as control. Absorbance at 550 nm was recorded. One unit of PAO activity was defined as the amount of enzyme required for catalyzing 1 μmol of Spd or Spm oxidation within 1 min.

### Determination of SOD, CAT, and NR Activity

Leaves were ground in 5 ml of 50 mM phosphate buffer (pH 7.8) and supernatants were recovered for determinations of SOD, CAT, and NR after centrifugation at 13,000 ×*g* for 15 min as previously described previously (Guo et al., 2006; Lu et al., 2014). SOD reaction solution (3 ml), which was consisted of 13 μM methionine, 1.3 μM riboflavin, 63 μM ρ-nitro blue tetrazolium chloride, and enzyme extract in 50 mM phosphate buffer (pH 7.8), was incubated for 10 min at room temperature under fluorescent light with 80 μmol m^-2^ s^-1^. Absorbance at 560 nm was determined with a spectrophotometer. One unit of SOD activity was defined as the amount of enzyme required for inhibition of photochemical reduction of ρ-nitro blue tetrazolium chloride (NBT) by 50%. CAT reaction that contained 15 mM H_2_O_2_ in 50 mM phosphate buffer (pH 7.0) was initiated by adding 50 μl of enzyme extract. The decreased absorbance at 240 nm within 1 min was recorded. One unit of CAT was defined as the amount of enzyme required for catalyzing the conversion of 1 μmol H_2_O_2_ within 1 min. NR reaction that contained 60 mM KNO_3_ and 0.25 mM NADH in 1.6 ml of 50 mM phosphate buffer (pH 7.5) was started by adding 0.4 ml of enzyme extract. After incubation at 25°C for 30 min, 1 ml of 1% sulfanilamide in 1.5 M HCl and 1 ml of 0.01% *N*-(1-naphthyl)-ethylenediammonium dichloride were immediately added into the reaction solution. Absorbance at 540 nm was recorded after centrifugation, and nitrite production was calculated by comparison with a standard curve of NaNO_2_. One unit of NR was defined as the amount of enzyme required for catalyzing the conversion of 1 mmol NO_2_ within 1 h.

### Determination of NO

Nitric oxide content was determined using Griess reagent as described previously (Zhou et al., 2005). Leaves (0.6 g) were ground in a mortar with pestle in 3 ml of 50 mM cool acetic acid buffer containing 4% zinc diacetate (pH 3.6). The homogenates were centrifuged at 10,000 × g for 15 min at 4°C. The supernatant was added by 0.1 g of activated charcoal. The filtrate was collected after vortex and filtration. One ml of filtrate was mixed with 1 ml of the Greiss reagent, followed by incubation for 30 min at room temperature. Absorbance at 540 nm was determined. NO content was calculated based on a standard curve of NaNO_2_.

### Determination of Protein Concentration

Protein concentration in enzyme extracts was determined using Coomassie Brilliant Blue G-250 solution using albumin from bovine serum (BSA) as a standard (Bradford, 1976).

### Statistical Analysis

The experiments were arranged in a completely randomized design with three pots of plants as replicates. For measurements of *A*, five leaves in each pot were randomly chosen and used for assay independently. For all the biochemical and physiological measurements, a pooled material from several different plants in each pot was randomly collected and used for assay. Significance of differences in the various parameters was assessed by one-way ANOVA (*P* < 0.05) using an SPSS program (SPSS, Inc., Chicago, IL, United States).

## Results

### Characterization of *CdSAMDC1*

**FIGURE 1:**
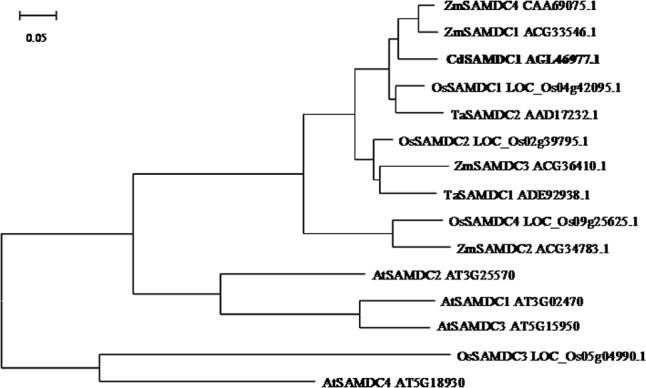
Phylogenetic analysis of CdSAMDC with SAMDCs in other plant species including rice (*Oryza sativa* L.), maize (*Zea mays* L.), wheat (*Triticum aestivum* L.), and Arabidopsis. The bar represents the branch length equivalent to 0.05 amino acid changes per residue.

**FIGURE 2:**
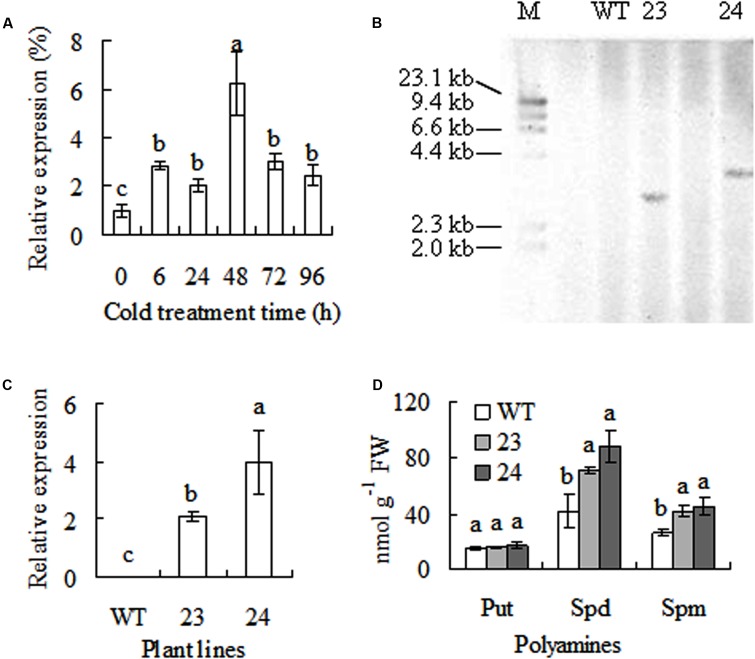
Response of *CdSAMDC1* transcript to cold treatment and analysis of transgenic centipedegrass (23, 24) overexpressing *CdSAMDC1* in comparison to the wild type (WT). Relative expression of *CdSAMDC1* in bermudagrass in response to cold (6°C) was determined by using real time quantitative RT-PCR (qRT-PCR, **A**). 20 μg of genomic DNA from transgenic centipedegrass and WT plants was digested with *Hind*III for DNA hybridization. **(B)** Relative expression of *CdSAMDC1* in transgenic centipedegrass was determined by using qRT-PCR using *actin* as reference. **(C)** Putrescine (Put), spermidine (Spd), and spermine (Spm) were determined by using HPLC **(D)**. Means of three repeats and standard errors are presented; the same letter above the column indicates no significant difference at *P* < 0.05. Put, Spd, and Spm levels were statistically analyzed separately.

A cDNA sequence of *CdSAMDC1* (GenBank accession number JX878505) with 1212-bp length was cloned from bermudagrass leaves. It encodes a peptide of 403 amino acids with 43.8 kDa and isoelectric point (pI) of 4.72. Sequence blast showed that CdSAMDC1 was most homologous (94%) to a CsSMADC2 (ADC45378) in *Cleistogenes songorica* in amino acid sequence, and 84 and 83% identical to ZmSAMDC4 in maize (CAA69075) and OsSAMDC1 in rice (LOC_Os04g42095), respectively. A phylogenetic analysis based on the full-length amino acid sequences showed that CdSMADC1 has a high identity with ZmSAMDC4, 1, and 2 (**Figure 1**). There is no insert in DNA sequence of *CdSAMDC1*. *CdSAMDC1* transcript was significantly induced after 6 h of cold treatment and reached to a maximum level at 48 h. Higher levels were maintained until to 96 h after cold treatment (**Figure 2A**).

### Polyamine Synthesis Was Improved in Transgenic Plants

Transgenic centipedegrass plants were molecularly detected. DNA hybridization signals by using *hpt* fragment as probe were shown in transgenic lines 23 and 24 with a unique integration pattern, while no signal was shown in the WT plants (**Figure 2B**). Compared to WT plants, *CdSAMDC1* transcript was detected in transgenic plants (**Figure 2C**). The results indicated that the transgene was integrated into centipedegrass genomes with one copy and *CdDAMDC1* was expressed in transgenic centipedegrass plants. Free polyamines were measured in transgenic centipedegrass in comparison with WT. Put level showed no difference between WT and transgenic plants. Compared to WT, Spd level was 2.3 to 2.9-fold higher and Spd level and 1.6 to 1.8-fold higher in transgenic lines (**Figure 2D**).

### Transgenic Plants Had Enhanced Cold Tolerance

**FIGURE 3:**
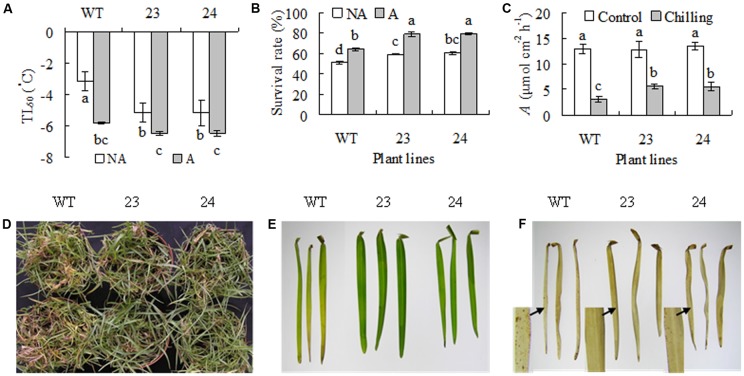
Analysis of freezing and chilling tolerance in transgenic plants in comparison with the WT. Plants were treated for 7 days at for cold acclimation **(A)**, followed by measuring ion leakage to calculate the temperature that resulted in 50% lethal (LT_50_, **A**) and survival rate after freezing treatment at –3°C **(B)**, while those were untreated by low temperature as non-acclimated control (NA). Net photosynthetic rate (*A*, **C**) were determined and photography **(D)** was taken after 30 days of chilling treatment at 6°C, followed by detaching the second leaf for taking photography **(E)** and H_2_O_2_ staining **(F)**. Means of three independent samples and standard errors are presented; the same letter above the column indicates no significant difference at *P* < 0.05.

Freezing tolerance was evaluated using LT_50_ and survival rate. Compared to a -3.2°C of LT_50_ in WT plants, transgenic lines had lower level of LT_50_ (-5.2°C) under non-acclimated condition. After 7 days of cold acclimation, LT_50_ decreased to -5.8°C in WT and -6.5°C in transgenic lines, respectively (**Figure 3A**). In consistence, WT had a 40% of survival rate, while transgenic lines had 59–61% of survival rate under non-acclimated condition. After cold acclimation, WT had a 64% of survival rate, while transgenic lines had a 79% of survival rate (**Figure 3B**). Chilling tolerance was also evaluated after 30 days of chilling treatment. WT and transgenic plants had similar *A* under non-stressed condition. Chilling treatment resulted in decrease in *A*, while significantly higher levels of *A* were observed in transgenic plants than in WT (**Figure 3C**). More brown or dead leaves were observed in WT than in transgenic plants after chilling treatment (**Figure 3D**). Color and H_2_O_2_ of the second leaf from the top were compared. Chilling treatment resulted in leaf browning in WT, but the leaf was maintained green in transgenic plants (**Figure 3E**). DAB staining showed that more H_2_O_2_ were accumulated in the second leaf in WT than in transgenic plants after chilling treatment (**Figure 3F**).

### Transgenic Plants Had Higher Antioxidant Enzyme Activities

**FIGURE 4:**
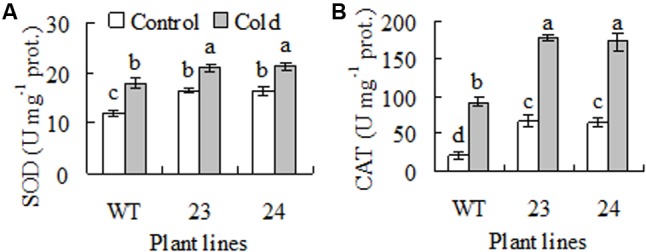
Superoxide dismutase (SOD, **A**) and catalase (CAT, **B**) activities in response to cold treatment in transgenic centipedegrass plants in comparison to the WT. The enzyme activities were measured from untreated control or cold (6°C for 7 days) treated plants. Means of three independent samples and standard errors are presented; the same letter above the column indicates no significant difference at *P* < 0.05.

Antioxidant enzymes protect plants against low temperature induced oxidative damage on photosynthetic apparatus and membrane system (Chen et al., 2013; Lu et al., 2013). Antioxidant enzyme activity was measured. Compared to WT, 37 to 39% and 2.1- to 2.2-fold higher activities of SOD and CAT were observed in transgenic plants under control condition. SOD and CAT activities were increased after 7 days of cold acclimation, and higher activities were still maintained in transgenic plant than in WT (**Figures 4A,B**).

### Transgenic Plants Had Higher PAO Activity and H_2_O_2_ Level

**FIGURE 5:**
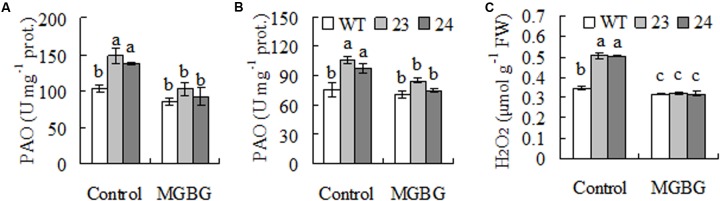
Polyamine oxidase (PAO) activity and H_2_O_2_ levels as affected by inhibitor of SAMDC in transgenic centipedegrass in comparison with the WT. PAO activity was measured after leaf fragments were pretreated for 12 h with MGBG, inhibitor of SMADC, using either Spd **(A)** or Spm **(B)** as substrate, while H_2_O_2_ was measured spectrophotometrically **(C)**. Means of three repeats and standard errors are presented; the same letter above the column indicates no significant difference at *P* < 0.05.

Spermindine and Spm are catabolized to produce H_2_O_2_ in plants catalyzed by PAO, while H_2_O_2_ is signaling in induction of cold responsive genes including those encoding antioxidant enzymes (Wan et al., 2009; Guo et al., 2014). Higher activity of PAO was observed in transgenic plants as compared with WT when either Spd or Spm was used as substrate (**Figures 5A,B**), and H_2_O_2_ level was higher in transgenic plants than in WT (**Figure 5C**). Feeding with MGBG, inhibitor of SAMDC, decreased PAO activity and H_2_O_2_ level in transgenic plants (**Figure 5**), indicating that the increased PAO activity and H_2_O_2_ level in transgenic plants were resulted from expression of *CdSAMDC1*.

### NR Dependent NO Production Is Involved in Elevated Antioxidant Enzyme Activities in Transgenic Plants

**FIGURE 6:**
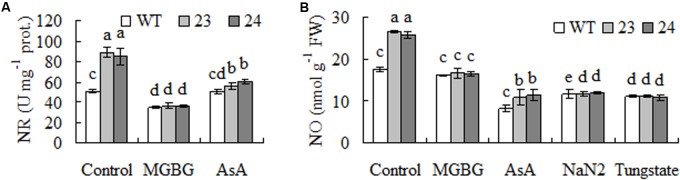
Nitrate reductase (NR) activity and nitric oxide (NO) level as affected by inhibitors of SAMDC and NR and scavenger of H_2_O_2_ in transgenic centipedegrass in comparison with the WT. NR activity **(A)** and NO concentration **(B)** were measured after leaf fragments were pretreated for 12 h. Means of three repeats and standard errors are presented; the same letter above the column indicates no significant difference at *P* < 0.05.

Hydrogen peroxide has been shown to induce expression of *NIR1* and lead to increased NR activity and accumulated NO in plants, while NO induces expression of antioxidant enzyme genes (Wan et al., 2009; Zhang et al., 2009; Lu et al., 2014). Higher levels of NR activities and NO were observed in transgenic plants compared with WT, which were blocked by pretreatment with AsA, an antioxidant to scavenge H_2_O_2_, or MGBG (**Figures 6A,B**), while pretreatment with NaN_2_ and tungstate, inhibitors of NR, inhibited the elevated NO in transgenic plants. The results indicated that the elevated NR and NO were associated with accumulation of H_2_O_2_ as a result of expression of *CdSAMDC1*, while the elevated NO was dependent upon NR.

**FIGURE 7:**
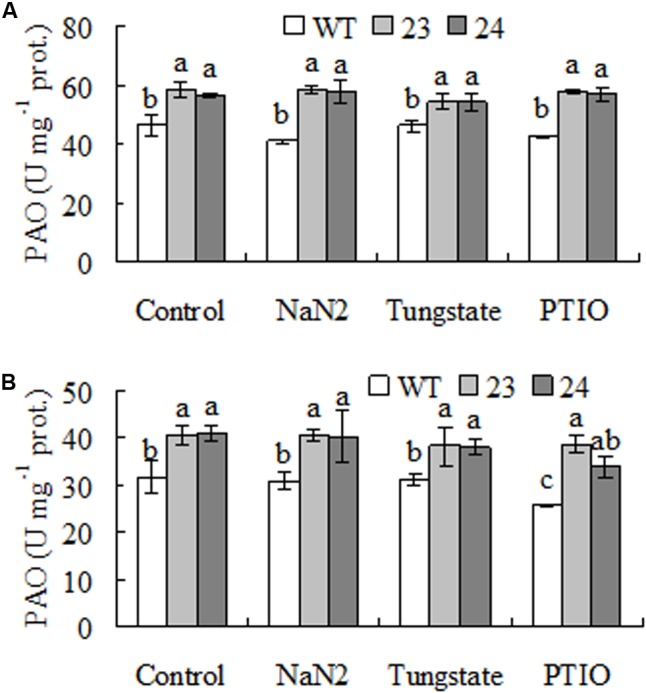
Polyamine oxidase (PAO) activity as affected by inhibitors of nitrate reductase (NR) and scavenger of NO in transgenic centipedegrass in comparison with the WT. Polyamine oxidase (PAO) activity was measured after leaf fragments were pretreated for 12 h using either Spd **(A)** or Spm **(B)** as substrate. Means of three repeats and standard errors are presented; the same letter above the column indicates no significant difference at *P* < 0.05.

To assess whether the elevated PAO activity was affected by NR dependent NO, PAO activity was measured after plants were treated by NR inhibitors and NO scavenger. The results showed that PAO activity was not altered by pretreatments with NaN_2_, tungstate, and PTIO, a scavenger of NO, when either Spd or Spm was used as substrate (**Figure 7**).

**FIGURE 8:**
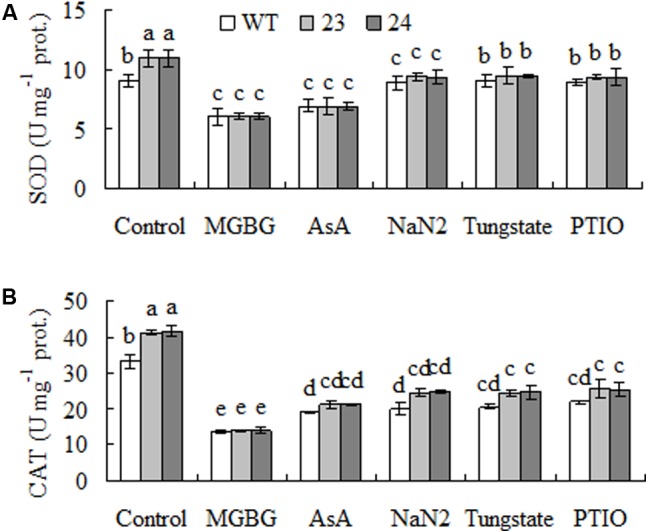
Superoxide dismutase (SOD, **A**) and catalase (CAT, **B**) activities as affected by inhibitors of SAMDC and nitrate reductase (NR) and scavenger of NO in transgenic centipedegrass in comparison with the WT. Enzyme activities were measured after leaf fragments were pretreated for 12 h. Means of three repeats and standard errors are presented; the same letter above the column indicates no significant difference at *P <* 0.05.

Activities of SOD and CAT were measured after pretreatment with MGBG, AsA, NaN_2_, tungstate, and PTIO to understand whether H_2_O_2_ and NO signaling were involved in the enhanced antioxidant enzyme activity in transgenic plants (**Figure 4**). Pretreatments with MGBG and AsA blocked the elevated activities of SOD and CAT in transgenic plants. Similarly, the elevated activities were also blocked by pretreatment with PTIO, NaN_2_ and tungstate (**Figures 8A,B**). The results indicated that the elevated SOD and CAT activities were associated with accumulated NO as a result of H_2_O_2_-dependent induction of NR.

## Discussion

A *SAMDC* gene, *CdSAMDC1*, showing induced expression by cold was isolated from bermudagrass in this study. CdSAMDC1 was highly identical to ZmSAMDC4, 1 in maize and OsSAMDC1 in rice. *OsSAMDC1* (Os04g42095) and *OsSAMDC4* (Os09g25625) are responsive to chilling among six SAMDCs in rice (Yamaguchi et al., 2004). Two transgenic centipedegrass lines overexpressing *CdSAMDC1* were generated in this study, with elevated levels of Spd in two lines and Spm in one line. Transgenic centipedegrass lines had higher survival rate and lower LT_50_ than WT under both cold acclimation and non-acclimation conditions, suggesting they had enhanced freezing tolerance. In addition, higher levels of *A* and less accumulation of ROS and dead leaves were observed in transgenic lines after 30 days of chilling treatment as compared with WT, suggesting that they had enhanced chilling tolerance. This is the first report on transgenic centipedegrass with expressing target gene and improved cold tolerance. Given that drought, salinity, cold and heat tolerance along with Spd and Spm levels can be altered by overexpressing either *SAMDC* or *SPDS* (Waie and Rajam, 2003; Kasukabe et al., 2004; Hao et al., 2005; Wi et al., 2006; Cheng et al., 2009), and higher levels of Spd and Spm are associated with chilling tolerance in a chilling-tolerant mutant of centipedegrass (Chen et al., 2013), our data indicated that the increased cold tolerance in transgenic centipedegrass was associated with the enhanced levels of Spd and Spm.

Polyamine oxidase catalyzes Spd and Spm oxidation to produce H_2_O_2_, while H_2_O_2_ is signaling in plant adaptation to stress and expression of cold responsive genes including those encoding antioxidant enzymes (Wan et al., 2009; Guo et al., 2014; Farnese et al., 2016; Niu and Liao, 2016; Sewelam et al., 2016; Singh et al., 2016). Compared to WT, higher activities of antioxidant enzymes and PAO were observed in transgenic centipedegrass plants. The elevated PAO activity was blocked by inhibitor of SAMDC, while the elevated antioxidant enzyme activities were blocked by inhibitor of SAMDC and scavenger of H_2_O_2_ in transgenic plants. The results suggest that the elevated PAO was associated with Spd and Spm synthesis, and the elevated antioxidant enzyme activities were dependent upon PAO-deprived H_2_O_2_. This case is supported by our previous observation that Spd and Spm induce PAO activity in tobacco (Guo et al., 2014). Higher antioxidant enzyme activities and transcripts were observed in transgenic tobacco plants overexpressing wheat *oxalate oxidase* which catalyzes oxalate oxidation to produce H_2_O_2_ (Wan et al., 2009; Lu et al., 2014). Antioxidant enzymes function to maintain homeostasis of ROS in plant cell by scavenging the accumulated ROS under stresses. Chilling-tolerant cultivars of centipedegrass, rice and *Stylosanthes guianensis* have higher antioxidant enzyme activities than sensitive cultivars (Guo et al., 2006; Chen et al., 2013; Lu et al., 2013). Nevertheless, the higher activities of antioxidant enzymes in transgenic centipedgrass were associated with the enhanced cold tolerance.

Nitrate reductase-dependent NO was demonstrated to be involved in H_2_O_2_ induced antioxidant enzyme activity in transgenic plants in this study. Our data showed that higher levels of NR activity and NO were observed in transgenic plants, which was blocked by inhibitor of SAMDC and scavenger of H_2_O_2_, suggesting that the elevated NR-deprived NO was dependent upon polyamine synthesis and H_2_O_2_. It was consistent with the case in bermudagrass and transgenic tobacco plants, in which H_2_O_2_ induces *NIR1* expression and results in higher NR activity and NO level (Lu et al., 2009, 2014). In addition, the increased activities of antioxidant enzymes in transgenic centipedegrass were blocked by inhibitors of NR and scavenger of NO, suggesting that the elevated antioxidant enzyme activities were dependent upon NR-deprived NO. Likely, the NR-dependent NO is involved in H_2_O_2_-induced antioxidant enzyme activities in transgenic tobacco and bermudagrass (Lu et al., 2009, 2014; Zhang et al., 2009). Exogenous treatment with H_2_O_2_ increased the NR-dependent NO level; either H_2_O_2_ or NO increases antioxidant enzyme activities in bermudagrass. The H_2_O_2_-induced antioxidant enzyme activities depend upon NO, but NO-induced antioxidant enzyme activities is not dependent upon H_2_O_2_ (Lu et al., 2009). Thus our results suggest that the improved Spd and Spm synthesis resulted in accumulation of PAO-deprived H_2_O_2_, which in turn led to higher levels of NR-dependent NO that increased antioxidant enzyme activities in transgenic centipedegrass plants.

In summary, overexpression of *CdSAMDC1* improved Spd and Spm synthesis in transgenic centipedegrass, which induced PAO activity for production of H_2_O_2_. The elevated H_2_O_2_ increased NR activity for production of NO, which in turn led to enhanced antioxidant enzyme activities and cold tolerance in transgenic plants.

## Author Contributions

JL and ML conducted most of the experiments of transgenic centipedegrass. CZ generated trangenic plants. PZ determined gene expression in bermudagrass. JC cloned CdSAMDC1 from bermudagrass. ZG and SL designed experiments and wrote manuscript.

## Conflict of Interest Statement

The authors declare that the research was conducted in the absence of any commercial or financial relationships that could be construed as a potential conflict of interest.

## Supplementary Material

FIGURE S1 Polyamine biosynthetic and oxidation pathway for Put, Spd, and Spm in plants. ADC, arginine decarboxylase; CPA, *N*-carbamoylputrescine amidohydrolase; ADI, agmatine deiminase; ODC, ornithine decarboxylase; CuAO, diamine oxidase; SAM, *S*-adenosylmethionine; SAMS, *S*-adenosylmethionine synthetase; SAMDC, SAM decarboxylase; dcSAM, decarboxylated *S*-adenosylmethionine; SPDS, spermidine synthase; SPMS, spermine synthase; PAO, polyamine oxidase.
