# Supplementary figures and images for: Transgenic Centipedegrass (Eremochloa ophiuroides [Munro] Hack.) Overexpressing S-Adenosylmethionine Decarboxylase (SAMDC) Gene for Improved Cold Tolerance Through Involvement of H2O2 and NO Signaling
Source: Front Plant Sci. 2017 Sep 22;8:1655. doi: 10.3389/fpls.2017.01655 (PMC5614975; doi:10.3389/fpls.2017.01655)

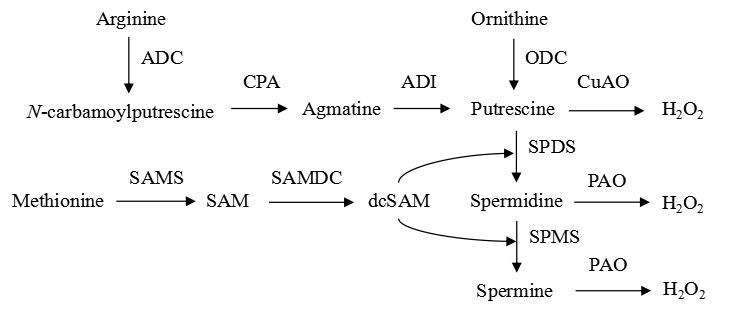

Supplement: FIGURE S1 — Polyamine biosynthetic and oxidation pathway for Put, Spd, and Spm in plants. ADC, arginine decarboxylase; CPA, N-carbamoylputrescine amidohydrolase; ADI, agmatine deiminase; ODC, ornithine decarboxylase; CuAO, diamine oxidase; SAM, S-adenosylmethionine; SAMS, S-adenosylmethionine synthetase; SAMDC, SAM decarboxylase; dcSAM, decarboxylated S-adenosylmethionine; SPDS, spermidine synthase; SPMS, spermine synthase; PAO, polyamine oxidase. [file Image_1.TIF]
